# Evaluating the normative implications of national and international artificial intelligence policies for Sustainable Development Goal 3: good health and well-being

**DOI:** 10.1093/haschl/qxaf108

**Published:** 2025-05-29

**Authors:** Francesca Mazzi

**Affiliations:** Brunel Law School, Brunel University London, Uxbridge UB8 3PH, United Kingdom

**Keywords:** artificial intelligence, sustainability, SDG 3, AI in healthcare, AI governance

## Abstract

**Introduction:**

Artificial intelligence (AI) has transformative potential in healthcare, promising advancements in diagnostics, treatment, and patient management, attracting significant investments and policy efforts globally. Effective AI governance, comprising guidelines, policy papers, and regulations, is crucial for its successful integration.

**Methods:**

This study evaluates 10 AI policies, namely focusing on 5 international organizations: the United Nations, the Organisation for Economic Co-operation and Development (OECD), the Council of Europe, the G20, and UNESCO, and 5 regional/national entities: Brazil, the United States, the European Union (EU), China, and the United Kingdom, to highlight the implications of AI governance for healthcare.

**Results:**

The EU AI Act focuses on risk management and individual protection while fostering innovation aligned with European values. The United Kingdom and the United States adopt a more flexible approach, offering guidelines to stimulate rapid AI integration and innovation without imposing strict regulations. Brazil shows a convergence toward the EU's risk-based approach.

**Conclusions:**

The study explores the normative implications of these varied approaches. The EU's stringent regulations may ensure higher safety and ethical standards, potentially setting a global benchmark, but they could also hinder innovation and pose compliance challenges. The United Kingdom's lenient approach may drive faster AI adoption and competitiveness but risks inconsistencies in safety and ethics. The study concludes by offering recommendations for future research.

## Introduction

Artificial intelligence (AI) holds transformative potential for achieving Sustainable Development Goal 3 (SDG 3), which aims to ensure healthy lives and promote well-being for all at all ages.^1^ Artificial intelligence technologies can significantly enhance healthcare delivery, improve disease prevention, and facilitate personalized medicine.^2^ For instance, AI-powered diagnostic tools can analyze medical images with high accuracy, enabling early detection of diseases such as cancer.^3^ Artificial intelligence algorithms can also predict outbreaks of infectious diseases by analyzing vast datasets, thereby enabling timely interventions.^4^ Moreover, AI can optimize healthcare resource allocation, ensuring that medical supplies and personnel are efficiently distributed to areas of greatest need.^5^ These capabilities align directly with the targets of SDG 3, which include reducing maternal and child mortality, combating epidemics, and achieving universal health coverage.^6^ By integrating AI into healthcare systems, we can address these targets more effectively, ultimately leading to improved health outcomes and enhanced quality of life globally.

### Ethical and legal considerations

The integration of AI into healthcare systems raises significant ethical and legal considerations that must be addressed to ensure that these technologies are deployed responsibly and equitably.^7^ Ethical concerns include the potential for bias in AI algorithms, which can lead to disparities in healthcare delivery and outcomes. For example, if AI systems are trained on datasets that lack diversity, they may not perform well for certain populations, exacerbating existing health inequalities.^8^ Privacy is another critical issue, as AI systems often require access to large amounts of personal health data.^9^ Such data are considered sensitive under the General Data Protection Regulation (GDPR) for example, which requires a high level of compliance for data controller and processor to ensure data protection.^10^ Ensuring the confidentiality and security of this data is paramount to maintaining patient trust. Legal considerations include the need for robust regulatory frameworks that govern the use of AI in healthcare, ensuring that these technologies meet safety and efficacy standards.^11^ At national level, countries are adopting different strategies in terms of policy and regulations in relation to AI. Some countries are developing general AI laws, while others are enacting sectorial regulations. In general, most countries have released policies with their visions on the concept of a “Good AI society,”^12^ but the effectiveness and enforceability of AI principles are still in its infancy.

### Relevance of AI governance for the integration of AI in healthcare

Artificial intelligence governance, encompassing guidelines, policy papers, and regulations, is critical for the development and integration of AI in healthcare. These regulatory frameworks ensure that AI applications are developed and deployed in a manner that prioritizes individuals (and therefore patients) safety, data privacy, and ethical considerations.^7^ Nationally, clear and comprehensive AI governance can facilitate the adoption of AI technologies by providing a structured environment that healthcare providers can trust.^13^ This regulatory clarity encourages investment in AI solutions and incentivizes innovation by setting predictable standards. For instance, policies that mandate rigorous testing and validation of AI systems before clinical use help prevent potential harm to patients and ensure the reliability of AI-driven diagnoses and treatments.

On an international level, AI governance is equally significant due to the scalable nature of many AI solutions.^8^ Technologies developed in one country can often be adapted and implemented in others, promoting global advancements in healthcare. However, this scalability raises legal and ethical considerations, such as data sharing across borders.^14^ International collaboration in AI governance can help facilitating the safe and effective global deployment of AI healthcare solutions.^15^ This research, which evaluates existing AI policies in relation to SDG 3 good health and well-being, aims to provide an understanding of how governments and international bodies position themselves regarding AI in healthcare.

## Methods

The purpose of the research was to analyze AI policies in relation to SDG 3, examining the extent to which these policies address health-related targets and indicators outlined in the SDG 3: Ensure healthy lives and promote well-being for all at all ages. This research is exclusively centered on general AI documents and policies, deliberately excluding sectorial regulations applicable to AI in healthcare, like the European Union (EU)'s medical device regulations or the Health Insurance Portability and Accountability Act (HIPPAA) in the United States. The rationale behind this focus lies in the observation that general AI documents often encapsulate the overarching vision of what constitutes an ideal AI-driven society. While sector-specific regulations undoubtedly play a crucial role in shaping AI's impact on healthcare, they may not fully capture the broader societal aspirations and ethical principles inherent in general AI policies.

The scope of the analysis was defined as follows:

- *Alignment with SDG 3 targets*: Evaluate whether AI policies explicitly address health-related targets and indicators outlined in SDG 3.
- *Integration of health considerations*: Examine the extent to which AI policies integrate health considerations into their objectives, strategies, and implementation plans.
- *Regulatory framework and ethical considerations*: Assess the adequacy and effectiveness of regulatory frameworks, guidelines, and ethical standards governing AI applications in relation to healthcare.

While these aspects can be evaluated to assess the alignment and potential impact of AI policies on health outcomes and SDG 3 targets, there are limitations to what can be feasibly evaluated. For example, assessing the long-term impact of AI policies may be challenging due to the complexity of health systems, the multifactorial nature of health outcomes, and the time lag between policy release and measurable health improvements. Contextual factors such as socio-economic conditions, cultural norms, political dynamics, and technological infrastructure may influence the effectiveness of AI policies in achieving health-related targets under SDG 3. Limiting the scope of analysis in a paper on the intersection of AI and health targets of SDG 3 due to a lack of structured empirical data is a common challenge faced by researchers in this field.^8^

### Selection of the AI policies

Limiting the analysis to certain AI policies and regulations worldwide is justified for feasibility reasons, primarily due to the vast and heterogeneous nature of global AI governance landscape. Analyzing every AI policy or regulation from every country and international organization would be impractical and resource intensive. The study focuses on 5 international organizations and 5 countries. The selection is composed of 5 international organizations, namely the United Nations, the Organisation for Economic Co-operation and Development (OECD), the Council of Europe, the G20, and UNESCO. As for the countries, establishing a selection criterion to identify policies from a representative sample of countries and international organizations is essential to ensure a manageable scope of analysis while capturing diversity in geographical and industrial development perspectives.

The selection criteria for identifying AI policies and regulations for analysis include the following:

- Geographical diversity;
- Industrial development perspective; and
- Policy maturity and impact; inclusion of international organizations.

Brazil, the United States, the EU, the People's Republic of China, and the United Kingdom were selected for analysis in this paper because they represent a diverse cross-section of global regions and have each played an active role in shaping AI governance. These jurisdictions offer valuable insights into a range of regulatory approaches, political systems, and economic contexts, thereby providing a broad comparative framework. While it is acknowledged that other countries have also enacted innovative and important AI-related policies, the scope of this paper necessitates a focused analysis. As such, the study is limited to these 5 jurisdictions to maintain depth and clarity of examination.

### Categorization of targets and indicators of SDG 3

To evaluate the extent to which AI policies address SDG 3, the SDG 3 targets and indicators were categorized in main themes based on the goals and means of implementation outlined within SDG 3. The main themes identified and selected for this research are maternal and child health, communicable diseases, noncommunicable diseases, substance abuse, environmental health, universal health coverage, and means of implementation, as presented in Table 1.

**Table 1.** SDG 3 targets and indicators themed.

Maternal and child health		Communicable diseases	Noncommunicable diseases	Substance abuse	Environmental health	Universal health coverage	Means of implementation:			
*Target 3.1: Maternal mortality*	*Target 3.2: Neonatal and child mortality*	*Target 3.3: Infectious diseases*	*Target 3.4: Noncommunicable diseases*	*Target 3.5: Substance abuse*	*Target 3.9: Environmental health*	*Target 3.8: Universal health coverage*	*Target 3.a: Tobacco control*	*Target 3.b: Medicines and vaccines*	*Target 3.c: Health financing and workforce*	*Target 3.d: Emergency preparedness*
Indicator 3.1.1: Maternal mortality ratio	Indicator 3.2.1: Under-5 mortality rate	Indicator 3.3.1: Number of new HIV infections per 1000 uninfected population	Indicator 3.4.1: Mortality rate attributed to cardiovascular disease, cancer, diabetes, or chronic respiratory disease	Indicator 3.5.1: Coverage of treatment interventions for substance use disorders	Indicator 3.9.1: Mortality rate attributed to household and ambient air pollution	Indicator 3.8.1: Coverage of essential health services	Indicator 3.a.1: Age-standardized prevalence of current tobacco use among persons aged 15 years and older	Indicator 3.b.1: Proportion of the target population covered by all vaccines included in their national program	Indicator 3.c.1: Health worker density and distribution	Indicator 3.d.1: International Health Regulations (IHR) capacity and health emergency preparedness
Indicator 3.1.2: Proportion of births attended by skilled health personnel	Indicator 3.2.2: Neonatal mortality rate	Indicator 3.3.2: Tuberculosis incidence per 100 000 population	Indicator 3.4.2: Suicide mortality rate	Indicator 3.5.2: Harmful use of alcohol	Indicator 3.9.2: Mortality rate attributed to unsafe water, unsafe sanitation, and lack of hygiene	Indicator 3.8.2: Proportion of population with large household expenditures on health as a share of total household expenditure or income				
		Indicator 3.3.3: Malaria incidence per 1000 population			Indicator 3.9.3: Mortality rate attributed to unintentional poisoning					
		Indicator 3.3.4: Hepatitis B incidence per 100 000 population								
		Indicator 3.3.5: Number of people requiring interventions against neglected tropical diseases								

Under maternal and child health, targets and indicators such as maternal mortality ratio, neonatal mortality rate, and under-5 mortality rate fall, aiming to reduce preventable deaths among mothers and children. Communicable diseases encompass targets related to ending epidemics of AIDS, tuberculosis, malaria, and neglected tropical diseases, as well as combating hepatitis and waterborne diseases. Noncommunicable diseases are addressed through targets focusing on reducing premature mortality from diseases like cardiovascular disease, cancer, diabetes, and chronic respiratory diseases.

Substance abuse is another critical theme, with targets aimed at strengthening prevention and treatment interventions for substance use disorders and harmful alcohol consumption. Environmental health targets aim to reduce deaths and illnesses from hazardous chemicals and pollution, including air, water, and soil contamination. Universal health coverage is a central theme, with targets focusing on ensuring access to essential health services, medicines, and vaccines for all individuals.

Finally, the means of implementation encompass targets related to tobacco control, research and development of medicines and vaccines, health financing and workforce development, and emergency preparedness. These targets aim to strengthen health systems, promote research and development, and build capacity to address global health challenges.

### AI policy analysis

To evaluate whether certain AI policies address the themes outlined in SDG 3 through textual analysis of policies, the research started by conducting a detailed textual analysis of AI policies at both national and international levels. The key policy objectives, goals, strategies, and measures outlined in the documents were identified. Then, the documents were scrutinized to find any references or mentions of health-related themes. Based on that, the research focused on evaluating the extent to which each policy aligns with the objectives and indicators of SDG 3 based on the thematic categorization and on assessing whether the policy content explicitly addresses health-related themes or indirectly contributes to improving health outcomes. The coherence and consistency of policy objectives with the overarching goals of SDG 3 were considered. Finally, a comparative methodology was adopted to make a critical evaluation of the differences between the selected AI policy frameworks.

## Results

The results of the analysis are presented in Table 2. The table shows the aspects of the analyzed AI policy frameworks that are relevant to the themed indicators and targets of SDG 3.

**Table 2.** AI policies analysis.

Relevant provisions for SDG 3?	The UN AI Advisory Body interim report	OECD	G20 guidelines	the UNESCO Recommendation on AI Ethics	the Council of Europe AI Treaty	Brazil	the US Executive Order on AI	The EU AI Act	The UK pro-innovation approach	People's Republic of China
	Yes	Yes	Indirect—Yes	Yes	Indirect—Yes	Yes	Yes	Yes	Yes	Yes
Maternal and child health	The report mentions AI's potential to improve maternal healthcare in regions like Sub-Saharan Africa. AI tools can enhance prenatal and postnatal care by providing better diagnostic tools and more personalized care plans, potentially reducing maternal and infant mortality rates	The OECD AI principles emphasise well-being and human-centric values, which can indirectly support maternal and child health by ensuring that AI applications in healthcare are developed and implemented responsibly. The focus on reducing inequalities and enhancing inclusion can support better healthcare access for mothers and children.	not specifically mentioned.	The document contains several provisions relevant to maternal and child health: Policy Area 6: Gender (Articles 87-93): Promotes gender equality and safety for girls and women, which indirectly supports maternal and child health by ensuring women's rights, safety, and empowerment in the digital age. Focuses on reducing gender gaps in various fields, including healthcare, and ensures women's representation and participation in AI-related fields. Addresses gender stereotyping and discrimination, advocating for the proactive redressal of biases in AI systems that could impact healthcare outcomes for women and children. Policy Area 11: Health and Social Well-being (Articles 121-124): Encourages the use of AI systems to improve human health, including maternal health, by mitigating disease outbreaks and ensuring the deployment of AI systems in healthcare aligns with international law and human rights obligations. Advocates for the inclusion of patients and their representatives in the development of health-related AI systems, ensuring these technologies are safe, effective, and efficient. Emphasizes oversight to minimize bias in health-related AI applications, ensuring privacy and informed consent, and maintaining human oversight in diagnosis and treatment decisions, which can significantly impact maternal and child health outcomes.	Not specifically mentioned.	The regulation emphasizes the protection of vulnerable groups, including children and adolescents, recognizing their aggravated vulnerability and ensuring their absolute priority (“proteção de grupos vulneráveis, em especial de idosos, de pessoas com deficiência e, com absoluta prioridade, de crianças e adolescentes, reconhecendo a vulnerabilidade agravada”).	The document does not explicitly address maternal and child health. However, the focus on improving healthcare delivery and the integration of AI in healthcare systems could indirectly benefit maternal and child health through enhanced diagnostics, predictive analytics, and better resource allocation.	The document references AI systems impacting healthcare services, including maternal care. For example, AI used to grant, reduce, revoke, or reclaim benefits and services like healthcare.	Not specifically mentioned.	The draft regulation mandates that AI systems should not provide minors with inappropriate content that may affect their physical and mental health, indicating a focus on protecting children's health
Communicable diseases	The report highlights AI's role in accelerating molecular and genomic research, which is crucial for developing treatments for communicable diseases. AI systems are being used for timely detection and diagnosis, which can be pivotal in managing diseases like tuberculosis and other infectious diseases	The principles encourage the use of AI to tackle global challenges, including health crises. AI can enhance early detection, tracking, and response to communicable diseases, contributing to better public health outcomes. Investment in AI research and development can spur innovations in managing and mitigating the spread of communicable diseases.	not specifically mentioned.	Policy Area 11: Health and Social Well-being (Articles 121-124): Emphasizes the role of AI in mitigating disease outbreaks, which is crucial for controlling communicable diseases. Highlights the importance of international solidarity in tackling global health risks and uncertainties, ensuring AI systems in healthcare are consistent with international laws and human rights. Calls for the development of prediction, detection, and treatment solutions for communicable diseases using AI, with careful attention to privacy, informed consent, and ethical considerations.	Not specifically mentioned.	Not specifically mentioned.	The executive order does not specifically mention communicable diseases. However, the enhancement of public health surveillance systems and the application of AI in monitoring and responding to health threats could play a significant role in managing and preventing outbreaks.	Not specifically mentioned.	Reference to AI applications in medical diagnostics and triage, which can enhance the identification and treatment of communicable diseases (eg, automated healthcare triage systems).	Not specifically mentioned.
Noncommunicable diseases	AI-powered diagnostic tools are mentioned as vital for early detection of noncommunicable diseases such as cancers and eye-related diseases. This early detection is essential for improving patient outcomes and reducing healthcare costs	The document's promotion of AI for well-being aligns with managing and treating noncommunicable diseases. AI-driven tools can help in monitoring, predicting, and personalizing treatment plans for chronic conditions.	not specifically mentioned.	Policy Area 11: Health and Social Well-being (Articles 121-124): Promotes the development and deployment of AI systems for health, including noncommunicable diseases, ensuring these technologies are scientifically and medically proven. Encourages evidence-based innovation and medical progress, involving patients and their representatives in the AI development process. Stresses the importance of bias mitigation, privacy, informed consent, and ethical oversight in AI systems used for healthcare, which can aid in the management and treatment of noncommunicable diseases.	Not specifically mentioned.	The regulation indirectly supports the management of noncommunicable diseases by promoting the ethical and responsible development and use of AI in healthcare contexts, which can be inferred from principles like “desenvolvimento e uso ético e responsável da IA”.	Not specifically mentioned.	The act emphasizes AI in healthcare, supporting diagnostic systems and human decision-making in health services, which would cover noncommunicable diseases as well.	Use of AI in medical devices and precision medicine, which can improve diagnostics and treatment of noncommunicable diseases.	Not specifically mentioned.
Substance abuse	While the report does not directly address substance abuse, the general principles of leveraging AI for health could extend to developing better intervention strategies and personalized treatments for substance abuse disorders.	Although not explicitly mentioned, AI's role in monitoring and predicting behaviors can be leveraged to identify and manage substance abuse cases more effectively, supporting preventive measures and personalized treatment plans.	not specifically mentioned.	Policy Area 11: Health and Social Well-being (Articles 121-124): While the document does not explicitly mention substance abuse, the principles of deploying AI in healthcare to improve health outcomes, mitigate risks, and ensure ethical oversight can be applied to addressing substance abuse issues. Ensures the involvement of healthcare professionals, patients, and ethical committees in developing AI systems, which can be tailored to create effective solutions for substance abuse prevention and treatment.	Not specifically mentioned.	Not specifically mentioned.	The executive order does not directly address substance abuse. The broader focus on healthcare improvement and the potential for AI to support mental health services might indirectly contribute to addressing substance abuse issues.	Not specifically mentioned.	Not specifically mentioned.	Not specifically mentioned.
Environmental health	AI's role in environmental health is discussed in the context of monitoring and predicting extreme weather events and managing biodiversity. These AI applications can indirectly support public health by addressing environmental determinants of health	The principle of protecting natural environments and promoting environmental sustainability directly supports the environmental health aspect of SDG 3. AI can contribute to better environmental monitoring and management, indirectly benefiting public health.	While environmental health is not directly discussed, the document emphasizes the need for a resilient and secure online environment which indirectly supports overall health including environmental factors through improved data handling and response systems.	Policy Area 10: Economy and Labour (Articles 116-120): Encourages assessing the impact of AI systems on labour markets, which includes considering the environmental implications of AI technologies. Advocates for interdisciplinary research, including the environmental impact of AI systems, ensuring sustainable development practices. Policy Area 11: Health and Social Well-being (Articles 121-124): While the focus is primarily on health, ensuring that AI systems are developed and deployed with attention to safety, efficiency, and ethical considerations indirectly supports environmental health by promoting sustainable and responsible AI practices.	While environmental health is not directly mentioned, the focus on sustainability and ethical AI indirectly supports healthier environments, which can benefit public health.	The regulation emphasizes the protection of the environment and the development of AI in an ecologically balanced manner (“proteção ao meio ambiente e ao desenvolvimento ecologicamente equilibrado”).	Environmental health is not explicitly covered in the document. However, AI applications in environmental monitoring and the healthcare sector could support initiatives to reduce pollution-related health risks and improve overall public health outcomes.	The act underscores the importance of environmental protection and highlights that AI systems should not have adverse impacts on fundamental rights, including the right to a high level of environmental protection.	References to AI's role in improving environmental health, such as through the development of plastic-eating enzymes and other sustainability technologies.	The regulation emphasizes green development, encouraging environmentally friendly and energy-saving technologies for AI development
**Universal health coverage**	Indirectly discusses unserved populations and calls for equitable AI innovation	Reduced disparities	There is an indirect link where the document mentions the need for inclusive, human-centric AI, which can support universal health coverage by making healthcare more accessible and efficient through digital transformation.	The ethical framework supports the reduction of health inequalities and the promotion of universal health coverage.	Not specifically mentioned.	Principles such as “crescimento inclusivo, desenvolvimento sustentável e bem-estar” and “direitos sociais, em especial a valorização do trabalho humano” support the broad goal of universal health coverage by ensuring that AI developments contribute to overall well-being and social rights.	Not specifically mentioned.	The document details AI's role in healthcare services, indicating a significant impact on access to universal health coverage by evaluating eligibility for public health benefits.	Indirectly addressed through the improvement of healthcare services and diagnostics via AI, which could contribute to more accessible and efficient healthcare.	The regulation emphasizes green development, encouraging environmentally friendly and energy-saving technologies for AI development
Means of implementation	Not specifically mentioned.	(targets not specifically mentioned). Indirect link: the principles outline the need for robust, secure, and safe AI systems, which are crucial for reliable healthcare applications. Accountability and transparency in AI systems ensure trust and reliability, critical for healthcare stakeholders and patients. The promotion of inclusive growth and reducing inequalities can lead to more equitable healthcare solutions, ensuring broader implementation of AI technologies across different demographics.	The document touches on the means of implementation by discussing the role of data and AI in enhancing digital infrastructure, ensuring fair and accountable use of AI, and promoting transparency and international cooperation. These elements are critical in implementing effective healthcare solutions.	Not specifically mentioned.	Not specifically mentioned.	Not specifically mentioned.	The document outlines comprehensive strategies for the development, deployment, and oversight of AI technologies in healthcare. This includes establishing AI safety programs, developing strategies for maintaining the quality of AI technologies, and ensuring compliance with federal nondiscrimination laws. Such measures support the sustainable implementation of AI in healthcare systems, aligning with the broader goals of SDG 3.	The act includes provisions on ensuring high-quality data governance and management practices, necessary for implementing effective AI systems in healthcare.	Not specifically mentioned.	The regulation includes comprehensive measures for AI governance, including data quality improvement, talent cultivation, industry-academia integration, and policy support for AI development, which are critical for the effective implementation of AI in healthcare
Aligned, neutral, or opposed?	The interim report aligns well with SDG 3 by promoting AI applications that enhance health outcomes across various dimensions. The emphasis on international collaboration, responsible sharing of AI technologies, and development of high-quality datasets reflects a commitment to using AI to achieve health-related goals globally. The report advocates for the equitable distribution of AI benefits, which is crucial for addressing health disparities and ensuring that advancements in AI contribute to global health equity.	The OECD AI principles are broadly aligned with SDG 3 (Good Health and Well-being). They emphasize the responsible use of AI to enhance well-being, reduce inequalities, and support sustainable development. By focusing on robustness, security, and safety, the principles ensure that AI applications in healthcare can be trusted and effectively integrated into health systems. The emphasis on transparency, human rights, and accountability ensures that AI technologies are used ethically and fairly, which is crucial for maintaining public trust and achieving health-related goals.	The policy is aligned with SDG 3 to the extent that it promotes inclusive, human-centric AI, fairness, transparency, and international cooperation. These principles can indirectly support various health-related goals by improving the efficiency, reach, and quality of healthcare services through digital transformation.	Aligned	the Council of Europe's policy document on AI aligns with SDG 3 through its broader commitments to ethical AI, human rights, and the Sustainable Development Goals (SDGs). Although there are no specific mentions of the health-related targets under SDG 3, the principles and frameworks suggested by the document support the creation of a regulatory environment where AI can be harnessed to improve health outcomes. The document emphasizes: Ethical and Human-Centered AI: This can contribute to better healthcare delivery and improved patient outcomes. Sustainability: Indirectly supports environmental health, which is crucial for overall public health. Equity and Inclusion: Ensuring that AI benefits all sections of society, including vulnerable populations, can enhance health equity.	Overall, Brazil's draft AI regulation aligns with SDG 3 by promoting the ethical, transparent, and accountable use of AI in healthcare. The regulation's focus on protecting vulnerable groups, including children and those with disabilities, and ensuring data privacy and security, supports the goal of ensuring healthy lives and promoting well-being for all ages. By mandating environmentally friendly practices, the regulation also contributes to a healthier environment, which is integral to SDG 3.	The executive order aligns with SDG 3 primarily through its emphasis on the responsible deployment and use of AI in healthcare, public health, and human services. By focusing on quality assurance, equity, and the mitigation of biases and risks associated with AI, the order promotes advancements in healthcare that could lead to improved health outcomes. The strategic plans and regulatory frameworks mandated by the order aim to ensure that AI technologies enhance healthcare delivery, public health surveillance, and patient safety, which are crucial components of SDG 3.	Overall, the EU AI Act aligns with SDG 3 by emphasizing the protection and improvement of health through AI systems. The act focuses on ensuring that AI systems used in healthcare are high-risk and must adhere to stringent requirements to safeguard health, safety, and fundamental rights. It promotes the use of AI in diagnostic systems, supporting healthcare decisions, and managing healthcare services. This alignment with SDG 3 ensures that AI systems contribute to health and well-being improvements, minimize harm, and support universal health coverage.	UK's AI policy document aligns with SDG 3 by promoting the use of AI to improve healthcare outcomes, enhance diagnostics, and support sustainable environmental practices. The document outlines the potential of AI in healthcare, particularly through automated triage systems and medical devices, which can improve the efficiency and accuracy of medical diagnoses and treatments. Additionally, the regulation emphasizes the importance of ethical considerations, data protection, and transparency in AI applications, which are crucial for maintaining public trust and ensuring equitable access to AI-driven healthcare innovations	China's draft AI regulation aligns with SDG 3 by promoting the use of AI in healthcare, ensuring data quality, and protecting vulnerable populations, including minors. The regulation's emphasis on green development and environmental health also indirectly supports SDG 3 by ensuring that AI technologies contribute to a healthier environment.
Normative implications	universal health coverage, AI to benefit of all humanity, particularly in vulnerable and underserved populations	disparity reduction, interdisciplinary and international cooperation, holistic approach to health including environmental sustainability	favour trust and international cooperation	direct support to the global agenda for health and well-being	legally binding treaty, focuses on sustainable, ethical and equitable development of AI.	protecting vulnerable populations and ensuring data privacy	focus on risks and biases, improved health outcomes, robust framework for integration of AI in healthcare.	, mandating high standards for AI systems that affect health and safety. By classifying healthcare-related AI systems as high-risk, the act enforces robust data governance, transparency, and risk management practices.	enhancing healthcare delivery and supporting public health initiatives	holistic vision, protection of child health, access to healthcare

Regarding international frameworks, the UN AI Advisory Body Interim Report highlights AI's potential to significantly improve maternal and child health, accelerate responses to communicable diseases, and support the diagnosis and management of noncommunicable diseases. The emphasis is on leveraging AI for social well-being and healthcare improvement globally. The OECD AI Principles: The OECD principles focus on promoting human well-being and inclusiveness. They encourage the use of AI to address global health challenges, although specific diseases are not directly mentioned. The principles advocate for the ethical use of AI in healthcare to enhance public health outcomes. The G20 guidelines acknowledge the indirect benefits of AI for health, although they do not specifically address SDG 3 themes. The guidelines focus more broadly on AI governance and ethical considerations, indirectly supporting health improvements through general AI advancements. The UNESCO Recommendation on AI Ethics contain several provisions related to health and well-being, explicitly addressing the potential of AI to improve health outcomes, including maternal and child health, and tackling noncommunicable diseases. It promotes ethical standards in AI to ensure it contributes positively to societal health. Finally, the Council of Europe AI Treaty mentions the relevance of AI to health indirectly, but it does not specifically address SDG 3 themes. The focus is on broader human rights and ethical considerations in AI deployment.

Regarding national frameworks, Brazil's AI regulation emphasizes the protection of vulnerable groups, including children and adolescents, indirectly supporting maternal and child health. It also supports the management of noncommunicable diseases through the ethical and responsible use of AI in healthcare contexts. The US Executive Order on AI does not specifically address the themes of SDG 3. It focuses on promoting AI innovation and ensuring the United States remains a leader in AI technology, with indirect implications for health through general advancements in AI.

The EU AI Act is more explicit in its health-related provisions, highlighting the role of AI in healthcare diagnostics and decision-making support. It recognizes AI's potential in managing noncommunicable diseases and improving healthcare services, aligning directly with SDG 3 goals. The UK Pro-Innovation Approach includes the use of AI in healthcare, particularly in diagnostics and medical devices, which can improve healthcare outcomes. The approach is flexible and focuses on encouraging innovation while ensuring safety and ethical standards. Finally, China's draft AI regulation mandates that AI systems protect vulnerable populations, including minors, indirectly supporting maternal and child health. It stresses the importance of responsible AI use in healthcare, although specific diseases are not mentioned.

The next 2 paragraphs present the normative implications of international and national frameworks analyzed.

### Normative implications of international frameworks of AI for SDG 3

#### The UN AI Advisory Body interim report, “Governing AI for humanity”

The normative implications of the UN AI Advisory Body Interim Report^16^ for SDG 3 are significant. The report underscores the necessity of embedding AI governance in international human rights frameworks and the SDGs, ensuring that AI developments contribute positively to global health. This approach promotes the ethical use of AI in healthcare, prioritizing inclusivity, equity, and the public interest. It also calls for robust international governance structures to oversee AI's deployment in health, ensuring that the technology is used safely, ethically, and for the benefit of all humanity, particularly in vulnerable and underserved populations.

#### The OECD recommendations on AI

The OECD AI principles^17^ establish a normative framework that supports the ethical and responsible use of AI in healthcare, aligning with the goals of SDG 3. By promoting transparency, accountability, and inclusivity, the principles encourage the development and deployment of AI systems that can enhance health outcomes and reduce disparities. The principles also underscore the importance of interdisciplinary research and international cooperation, which are vital for addressing global health challenges. The commitment to environmental sustainability further reinforces the interconnected nature of health and the environment, advocating for holistic approaches to health that consider broader ecological impacts.

#### The G20 guidelines on AI

The G20 principles on AI lay the groundwork for ethical, accountable, and transparent use of AI technologies. These principles are crucial for the healthcare sector, which is increasingly reliant on AI to enhance patient care, diagnostics, and operational efficiencies. The normative implications of this policy for SDG 3 include the establishment of standards that ensure AI systems respect privacy, promote equality, and operate transparently. This fosters trust in AI-driven healthcare solutions, which is essential for broad acceptance and effective implementation.

By promoting international cooperation and fair practices, the policy supports the creation of global standards for AI in healthcare, facilitating cross-border data flows and collaborative research. These efforts can accelerate the development and deployment of AI solutions tailored to address critical health issues, such as disease outbreaks, chronic disease management, and health resource allocation. The policy's emphasis on ethical AI use and robust governance frameworks helps ensure that AI technologies contribute positively to the goal of good health and well-being for all, in alignment with SDG 3.

#### The UNESCO recommendations on AI

The UNESCO recommendations on AI^18^ carry significant normative implications for achieving SDG 3, which aims to ensure healthy lives and promote well-being for all at all ages. The recommendations advocate for the integration of AI systems in healthcare, emphasizing the potential of these technologies to improve health outcomes, mitigate disease outbreaks, and enhance patient care. By encouraging the development and deployment of AI in ways that are safe, effective, and ethically sound, the guidelines align with SDG 3's targets on maternal and child health, communicable and noncommunicable diseases, substance abuse, and environmental health. They stress the importance of maintaining human oversight in health-related AI applications, ensuring patient consent and privacy, and addressing biases within AI systems. This ethical framework supports the reduction of health inequalities and the promotion of universal health coverage, thereby advancing the global agenda for health and well-being. Moreover, the focus on gender-responsive AI policies, as highlighted in Policy Area 6, ensures that AI advancements contribute to reducing gender disparities in health, further reinforcing the commitment to equitable health outcomes.

#### The AI treaty of the Council of Europe

The normative implications of the Council of Europe's treaty on AI^19^ for SDG 3 center around promoting ethical, equitable, and sustainable AI development and deployment. By advocating for human-centered AI, the document implicitly supports initiatives that can improve health outcomes. The focus on ethical standards and human rights aligns with the goals of ensuring healthy lives and promoting well-being for all at all ages. While the document does not explicitly address specific health issues, its broader principles foster an environment where AI can be effectively utilized to support and enhance public health initiatives.

### Normative implications of national frameworks of AI for SDG 3

#### The Brazilian draft legislation on AI

Brazil has re-introduced a proposal for an AI legislation.^20,21^ The normative implications of Brazil's draft AI regulation for SDG 3 are substantial. The regulation establishes a framework for the responsible development and use of AI, ensuring that AI systems enhance health outcomes without compromising ethical standards. The emphasis on protecting vulnerable populations and ensuring data privacy aligns with the core principles of SDG 3. Additionally, the regulation promotes transparency, accountability, and human oversight, which are critical for maintaining public trust in AI technologies. By addressing the potential risks and ensuring robust governance, the regulation supports the sustainable and equitable integration of AI in healthcare, contributing to the overall goal of achieving universal health coverage and improved health and well-being for all.

#### The US Executive Order on AI

In January 2025, Trump signed a new executive order on AI. The Executive Order on Removing Barriers to American Leadership in Artificial Intelligence (January 23, 2025) revokes certain existing AI policies and directives perceived as barriers to American AI innovation. It aims to clear a path for the United States to act decisively to retain global leadership in AI. The order mandates the creation of an action plan within 180 days to sustain US AI leadership, focusing on human flourishing, economic competitiveness, and national security. This order is intended to foster AI innovation across various sectors, including healthcare, by promoting AI integration and reducing regulatory hurdles.

#### The EU AI Act

The normative implications of the EU AI Act^22^ for SDG 3 are significant. The act sets a precedent for the responsible use of AI in healthcare, mandating high standards for AI systems that affect health and safety. By classifying healthcare-related AI systems as high risk, the act enforces robust data governance, transparency, and risk management practices. This ensures that AI applications in healthcare are safe, reliable, and equitable, addressing potential biases and protecting vulnerable individuals. The act promotes trust in AI systems, which is crucial for their acceptance and effective implementation in healthcare. Thus, it supports the overarching goal of SDG 3 by ensuring healthy lives and promoting well-being for all at all ages through the ethical and safe use of AI technology.

#### The People's Republic of China draft AI law

The normative implications of the preliminary draft of China's proposed AI Law that has circulated among legal scholars^23^ for SDG 3 are profound. By mandating high standards for AI in healthcare and emphasizing ethical use, the regulation ensures that AI technologies enhance health outcomes while protecting individual rights. The focus on data quality, transparency, and accountability is crucial for building trust in AI systems, which is essential for their widespread adoption in healthcare. The regulation's provisions for protecting minors and promoting green development highlight a holistic approach to health that encompasses both direct medical care and broader environmental and social determinants of health. This comprehensive approach supports the goal of ensuring healthy lives and promoting well-being for all ages as outlined in SDG 3.

#### The UK pro-innovation approach

The UK pro-innovation approach to AI^24^ aligns with SDG 3 by promoting the use of AI to improve healthcare outcomes, enhance diagnostics, and support sustainable environmental practices. The document outlines the potential of AI in healthcare, particularly through automated triage systems and medical devices, which can improve the efficiency and accuracy of medical diagnoses and treatments. This has significant normative implications for SDG 3, particularly in terms of enhancing healthcare delivery and supporting public health initiatives. By prioritizing the ethical use of AI, the policy aims to mitigate risks associated with AI deployment, such as data privacy concerns and potential biases in healthcare decisions. The focus on transparency, accountability, and human oversight ensures that AI systems are implemented in a manner that promotes health equity and patient safety.

## Discussion

The normative implications of the analyzed AI policy frameworks for achieving SDG 3 are substantial. A critical comparison reveals significant differences between international frameworks and national policies in terms of scope, enforceability, and their impact on health outcomes.

International frameworks, such as the UN AI Advisory Body Interim Report, the OECD AI principles, and the UNESCO Recommendations on AI Ethics, provide broad guidelines emphasizing the ethical, transparent, and accountable use of AI. These frameworks aim to align AI development with global health goals by promoting inclusivity, equity, and the public interest. They encourage interdisciplinary research and international cooperation, which are crucial for addressing global health challenges. However, these international guidelines often lack enforceability, relying instead on moral suasion and the voluntary adoption of best practices by nations and organizations.

In contrast, national frameworks, such as the EU AI Act, Brazil's draft AI regulation, and China's proposed AI law, introduce specific, enforceable regulations that directly impact healthcare.^25^ The EU AI Act, for instance, classifies healthcare-related AI systems as high risk, mandating stringent data governance, transparency, and risk management practices.^26^ This regulatory approach ensures that AI applications in healthcare are safe, reliable, and equitable, addressing potential biases and protecting vulnerable populations. The enforceability of such regulations within the EU, and potentially beyond under certain conditions,^27^ offers robust guarantees for health, promoting trust and widespread adoption of AI in healthcare.

The UK and US approaches, on the other hand, are more flexible and pro-innovation, focusing on creating an environment that fosters AI development while addressing risks through non-binding guidelines.^28^ The United Kingdom's pro-innovation approach emphasizes the potential of AI to enhance healthcare delivery through automated triage systems and advanced diagnostics, while promoting ethical use, transparency, and human oversight. This approach aims to balance the need for innovation with the mitigation of risks such as data privacy concerns and potential biases in AI systems.

One of the key advantages of stringent regulations, such as those in the EU AI Act, is the high level of assurance they provide in terms of safety, ethical standards, and data protection. These regulations help build public trust in AI technologies, which is essential for their effective implementation in healthcare. However, the downside is that such rigorous requirements can potentially stifle innovation by imposing significant compliance burdens on developers and businesses.

Conversely, the more flexible, principle-based frameworks seen in the United Kingdom and the United States encourage rapid innovation and adaptation of AI technologies. This can lead to faster advancements and integration of AI in healthcare, potentially driving significant improvements in health outcomes. The trade-off, however, is the risk of insufficient oversight, which can lead to issues such as data privacy breaches, biased algorithms, and unequal access to AI-driven healthcare solutions.

### AI governance and health equity

Artificial intelligence governance can significantly influence health equity by ensuring that AI technologies are developed and used in ways that are inclusive and equitable.^29^ Strong governance frameworks can help address the disparities in healthcare access and quality that exist within and between countries. For instance, governance frameworks that focus on data privacy, bias mitigation, and transparency can prevent the exclusion of marginalized groups and incentivize an equitable distribution of AI benefits.^30^ The UNESCO recommendations, with their focus on gender-responsive AI policies, exemplify how governance can reduce gender disparities in health.

Furthermore, governance that prioritizes the protection of vulnerable populations, as seen in Brazil's draft AI legislation, can enhance health equity by ensuring that AI systems do not harm or exclude these groups. However, countries face diverse challenges in implementing AI governance for healthcare. In developed nations like those in the EU, the primary challenge lies in balancing stringent regulatory standards with the need to foster innovation. While robust regulations like the EU AI Act provide high levels of assurance for safety and ethical use, they can also create significant compliance costs and potentially slow down innovation, as mentioned before. Ensuring that regulations are flexible enough to adapt to rapid technological advancements is crucial.

### Challenges of implementing AI policies in healthcare settings

Implementing AI policies in healthcare settings involves a number of practical, legal, and ethical challenges that can significantly hinder effective deployment. On a technical level, issues related to data quality, privacy, and interoperability remain central and already visible in relation to data protection legislation and how it creates a delicate trade-off.^31^ Artificial intelligence systems require large, representative datasets, yet many healthcare datasets are fragmented, biased, or lack proper anonymization—leading to skewed outcomes and risks to patient confidentiality.^32^ Clinically, the integration of AI into existing workflows often faces resistance from healthcare professionals who are either undertrained in AI tools or concerned about the erosion of clinical autonomy, misdiagnosis risks, and unclear lines of liability.^33^ Legally, the lack of clear, harmonized regulation on AI accountability, informed consent for algorithm use, and the classification of AI as a medical device complicates compliance and oversight across jurisdictions.^34^ Moreover, ethical concerns such as algorithmic bias, transparency, and fairness pose systemic risks that may exacerbate existing healthcare inequalities if not addressed through inclusive governance frameworks.^35^ Institutional capacity is another challenge—many healthcare providers, particularly in low-resource settings, lack the infrastructure and funding to safely implement and maintain AI systems. Finally, cybersecurity risks and data breaches represent growing concerns, requiring robust protocols to protect sensitive health information.^36^ Addressing these multifaceted challenges will require interdisciplinary collaboration, adaptive regulation, and sustained investment in digital health literacy.

### Recommendations for future research on AI governance and SDG 3

Future research should focus on several key areas to ensure that AI governance effectively supports the dual goals of innovation and health equity.

Firstly, given the dynamic nature of AI governance, it would be desirable to continue monitoring new and existing AI governance frameworks at both national and international levels with comparative methods. Comparative studies should assess the effectiveness of stringent regulatory frameworks, such as the EU AI Act, against more flexible, principle-based approaches, like the United Kingdom's pro-innovation strategy. Evaluating the impact of these frameworks on innovation, public trust, and the distribution of AI benefits in healthcare is also essential to identify best practices and areas for improvement. As shown by recent research, Americans for example seem to perceive healthcare as an area in which AI applications could be particularly beneficial, but they have substantial concerns regarding specific applications, especially those in which AI is involved in decision-making and regarding the privacy of health information.^37^

Another area of research should explore how AI governance frameworks impact health equity and the ways in which AI governance can promote inclusive access to AI technologies, particularly in underserved and marginalized communities.^38^ Building public trust in AI technologies is another critical area of research. This includes developing standards for how AI decisions are communicated to patients and healthcare providers.^39^ Research should also examine the role of AI governance in mandating monitoring of quality of AI in healthcare.^40^

Interdisciplinary and international collaboration is key to developing effective AI governance frameworks that can maximize the positive outcome on AI in healthcare as a consequence. Future research should focus on studying the role of international organizations such as the World Health Organization and the International Telecommunication Union in setting standards. Exploring the benefits of cross-sector partnerships between governments, academia, industry, and civil society in developing and implementing AI governance frameworks can provide valuable case studies and lessons learned.

Also, future research should track health outcomes in regions and institutions that have implemented AI governance frameworks, measuring improvements in health indicators, patient satisfaction, and system efficiencies. Analyzing the economic impact of AI implementation in healthcare, including cost savings, return on investment, and economic disparities, can help quantify the benefits and costs associated with AI adoption^41^ and identify eventual differences under different governance models.

While this paper offers a comprehensive analysis of AI governance through policy review and theoretical frameworks, it does not include empirical data assessing the practical outcomes of these regulatory approaches—particularly in terms of their impact on health-related applications of AI. This limitation is acknowledged and presents a valuable opportunity for future research. The conceptual groundwork laid here can serve as a foundation for a follow-up empirical study that investigates how these policies are implemented in practice and their tangible effects on public health systems, equity, and innovation. Future research should aim to gather qualitative and quantitative data from stakeholders, including policymakers, healthcare providers, and affected communities, to evaluate the real-world efficacy and consequences of AI governance across jurisdictions.

## Conclusions

The analysis of national and international AI frameworks highlights the critical role of governance in shaping the development and deployment of AI technologies in healthcare. These frameworks vary significantly in their approach, enforceability, and impact, offering precious insights into how different regions are addressing the ethical, legal, and social challenges associated with AI in healthcare.

The relevance of these frameworks for SDG 3 lies in their ability to shape the ethical landscape of AI in healthcare, ensuring that technological advancements contribute positively to health and well-being. Strong governance frameworks that prioritize equity, inclusivity, and ethical standards are fundamental for mitigating risks and maximizing the benefits of AI in healthcare.

International frameworks provide valuable ethical guidelines and promote global cooperation, essential for addressing shared health challenges and ensuring that AI benefits are equitably distributed. However, their impact is limited by their non-binding nature (except for the AI treaty), highlighting the importance of national frameworks that can enforce these principles.

National frameworks, being them policies and/or draft regulations, play a critical role in ensuring the safe, reliable, and equitable deployment of AI in healthcare. How to achieve the balance between stringent regulations and flexible, innovation-friendly policies is a key question mark.

## Supplementary Material

qxaf108_Supplementary_Data
